# Connectionism coming of age: legacy and future challenges

**DOI:** 10.3389/fpsyg.2014.00187

**Published:** 2014-03-04

**Authors:** Julien Mayor, Pablo Gomez, Franklin Chang, Gary Lupyan

**Affiliations:** ^1^Department of Psychology and Educational Sciences, University of GenevaGenève, Switzerland; ^2^Department of Psychology, De Paul UniversityChicago, IL, USA; ^3^Department of Psychological Sciences, University of LiverpoolLiverpool, UK; ^4^Department of Psychology, University of WisconsinMadison, WI, USA

**Keywords:** recurrent networks, interactive processing, probabilistic cognition, computational modeling, language acquisition, language processing, speech perception, computational linguistics

## About 50 years after the introduction of the perceptron and some 25 years after the introduction of PDP models, where are we now?

In 1986, Rumelhart and McClelland took the cognitive science community by storm with the Parallel Distributed Processing (PDP) framework. Rather than abstracting from the biological substrate as was sought by the “information processing” paradigms of the 1970s, connectionism, as it has come to be called, embraced it. An immediate appeal of the connectionist agenda was its aim: to construct at the algorithmic level models of cognition that were compatible with their implementation in the biological substrate.

The PDP group argued that this could be achieved by turning to networks of artificial neurons, originally introduced by McCulloch and Pitts (1943) which the group showed were able to provide insights into a wide range of psychological domains, from categorization, to perception, to memory, to language. This work built on an earlier formulation by Rosenblatt (1958) who introduced a simple type of feed-forward neural network called the perceptron. Perceptrons were limited to solving simple linearly-separable problems and although networks composed of perceptrons were known to be able to compute any Boolean function (including XOR, Minsky and Papert, 1969), there was no effective way of training such networks. In 1986, Rumelhart, Hinton and Williams introduced the back-propagation algorithm, providing an effective way of training multi-layered neural networks, which could easily learn non linearly-separable functions. In addition to providing the field with an effective learning algorithm, the PDP group published a series of demonstrations of how long standing questions in cognitive psychology could be elegantly solved using simple learning rules, distributed representations, and interactive processing.

To take a classic example, consider the word-superiority effect, in which people can detect letters within a word faster than individual letters or letters within a non-word (Reicher, 1969). This result is difficult to square with serial “information-processing” theories of cognition that were dominant at the time (how could someone recognize “R” before “FRIEND” if recognizing the word required recognizing the letters?). Accounting for such findings demanded a framework which could naturally accommodate interactive processes within a bidirectional flow of information. The so-called “Interactive-activation model” (McClelland and Rumelhart, 1981) provided just such a framework.

The connectionist paradigm was not without its critics. The principal critiques can be divided into three classes. First, some neuroscientists (Crick, 1989) questioned the biological plausibility of backpropagation, when they failed to observe experimentally complex and differentiated back-propagating signals that are required to learn in multi-layered neural networks. A second critique concerned stability-plasticity of the learned representations in these models. Some phenomena require the ability to rapidly learn new information, but sometimes newly learned knowledge overwrites previously learned information (catastrophic interference; McCloskey and Cohen, 1989). Third, representing spatial and temporal invariance—something that apparently came easily to people—was difficult for models, e.g., recognizing that the letter “T” in “TOM” was the “same” as the “T” in “POT.” This invariance problem was typically solved by multiplying a large number of hard-wired units that were space- or time-locked (see e.g., McClelland and Elman, 1986). Finally, critics pointed out that the networks were incapable of learning true rules on which a number of human behavioral, namely language-learning was thought to depend (e.g., Marcus, 2003; cf. Fodor and Pylyshyn, 1988; Seidenberg, 1999).

The connectionist approach has embraced these challenges: Although some connectionist models continue to rely on backpropagation, others have moved to more biologically realistic learning rules (Giese and Poggio, 2003; Masquelier and Thorpe, 2007). Far from being a critical flaw of connectionism, the phenomenon of catastrophic interference (Mermillod et al., 2013) proved to be a feature that led to the development of complementary learning systems (McClelland et al., 1995).

Progress has also been made on the invariance problem. For example, within the speech domain representing the similarity between similar speech sounds regardless of their location within a word has been addressed in the past by Grossberg and Myers (2000) and Norris (1994) and this issue presents a new more streamlined and computationally efficient model (Hannagan et al., 2013). An especially powerful approach to solving the location invariance problem in the visual domain is presented by Di Bono and Zorzi (2013), also in this issue.

A key challenge for connectionism is to explain the learning of abstract structural representations. The use of recurrent networks (Elman, 1990; Dominey, 2013) and self-organizing maps, has captured important aspects of language learning (e.g., Mayor and Plunkett, 2010; Li and Zhao, 2013), while work on deep learning (Hinton and Salakhutdinov, 2006) has made it possible to model the emergence of structured and abstract representations within multi-layered hierarchical networks (Zorzi et al., 2013). The work on verbal analogies by Kollias and McClelland (2013) continues to address the challenges of modeling more abstract representations, but truly understanding how neural architectures give rise to symbolic cognition is a gap that remains. Although learning and representing formal language rules may not be completely outside of the abilities of neural networks (e.g., Chang, 2009), it seems clear that understanding human cognition requires understanding how we solve these symbolic problems (Clark and Karmiloff-Smith, 1993; Lupyan, 2013). Future generations of connectionist modelers may wish to fill this gap and in so doing provide a fuller picture of how neural networks give rise to intelligence of the sort enables us to ponder the very workings of our cognition.

## What's next?

The articles assembled in this issue demonstrate the range of topics currently addressed by connectionist models: from word learning in atypical populations (Sims et al., 2013), to sentence processing (Hsiao and MacDonald, 2013), to multimodal processing (Bergmann et al., 2013), to interactions between language and vision (Smith et al., 2013). We expect this diversity to continue to increase. We also hope to see greater increasing integration between connectionism and a computationally similar but philosophically distinct models employing Bayesian inference. Although the computational similarities between these two approaches have been previously recognized (McClelland, 1998), detailed tutorials like the one contained in this volume (McClelland, 2013) provide new clarity on the relationship between these two approaches.

The influence of theoretical constructs introduced by the connectionist approach have become part and parcel of cognitive science (although they are now often not accompanied by the label “connectionism” or “PDP”). The distributed representations that challenge classical symbolic models and which emerge naturally in neural networks are now no longer theoretical constructs and can be directly observed in the brain (Kriegeskorte et al., 2008; Chang et al., 2010). Evidence for rapid warping of these representations by task demands (of the sort described by e.g., McClelland and Rogers, 2003) is also being confirmed through modern neuroimaging (e.g., Çukur et al., 2013)^1^. Many connectionist models have stressed prediction as a way of learning structure and statistical inputs (e.g., Dell and Chang, 2014). This too finds wide support in contemporary neuroscience (Friston, 2010) leading some to even argue that prediction is the unifying feature of all cognitive and perceptual processes (Clark, 2013, for review). Interactive processing—another core feature of the connectionist paradigm—has become similarly foundational. The interplay between bottom-up and top-down information is now recognized to be critical from everything as simple as simply detecting the presence of a visual stimulus, to consciousness itself (e.g., Dehaene et al., 2003; Gilbert and Sigman, 2007; Lupyan and Ward, 2013).

Finally, contemporary neural networks, most notably those utilizing so called deep-learning, have found success in solving practical problems such as image and speech recognition, and natural language processing. For example, algorithms based on the deep-learning approach are now used by Google to extract high-level features from images with, in some cases, above-human performance (Ciresan et al., 2011; Le et al., 2011).

## Footnotes

^1^It is useful to note that the methods that make these analyses possible, most notably multi-voxel pattern analyses (MVPA, e.g., Norman et al., 2006) and “representational dissimilarity matrices” (Kriegeskorte et al., 2008) are adaptations of methods developed for analyzing dynamics of artificial neural networks.
